# The effectiveness of HPV vaccination on the incidence of oropharyngeal cancers in men: a review

**DOI:** 10.1186/s13027-022-00479-3

**Published:** 2023-04-24

**Authors:** Philip Macilwraith, Eve Malsem, Sathana Dushyanthen

**Affiliations:** 1 The Prince Charles Hospital, 627 Rode Road, Chermside, QLD 4032 Australia; 2 Ballarat Base Hospital, 1 Drummond St North, Ballarat Central, VIC 3350 Australia; 3 University of Melbourne, Parkville, VIC 3010 Australia

**Keywords:** HPV, Oropharyngeal cancer, Vaccine, Vaccination, Boys, Male, Review, Pangender HPV vaccination, Head and neck cancer

## Abstract

**Background:**

Human Papilloma Virus (HPV) contributes to the development of oropharyngeal cancer (OPC) and is currently the leading cause of OPC in the Western world. There have been limited studies examining the effect of HPV-vaccination on OPC incidence in men. This review aims to interrogate relationship linking HPV-vaccination and OPC in men, to potentially recommend pangender HPV-vaccination, to reduce the incidence of HPV associated OPC.

**Main Body:**

A review was carried out using Ovid Medline, Scopus and Embase databases, on 22nd October 2021 investigating the effect of HPV-vaccination on OPC prevalence in men and including studies with vaccination data pertaining to men in the past 5 years, while excluding those studies without appropriate oral HPV-positivity data and non-systematic reviews. Studies were evaluated as per the PRISMA guidelines and ranked using risk of bias tools including RoB-2, ROBINS-1 and the NIH quality assessment tools. 7 studies were included ranging from original research to systematic review articles. All studies were published in English from 2017 to 2021. Overall, these suggested that HPV-vaccination reduced levels of oral HPV positivity in men. This was thought to be indicative of a reduced risk of development of HPV-associated OPC. A limitation of this study was the inability to conduct meta-analysis due to the heterogeneity of included studies. We noted a significant impact on the reduction of HPV positivity post HPV-vaccination and a potential contribution to reducing the future incidence of OPC.

**Conclusion:**

This review makes a strong case for pangender HPV-vaccination in combatting OPC in men.

## Background

### Oropharyngeal Cancer

The prevalence and causes of oropharyngeal cancer (OPC) have altered significantly over the past 20 years. As tobacco use declines, particularly in Western countries, Human Papilloma Virus (HPV) has now become the main risk factor for OPC, accounting for more than 70% of cases in the United States [1]. In comparison, the relative rates of Australian HPV-associated OPC have risen from 0.2% in 1995 to over 63.2%, as reported by Hong et al., 2015 [2]. OPC has overtaken cervical cancer as the main cause of HPV-related malignancy [1]. In contrast to cervical cancer, HPV-associated OPC does not have an identifiable precursor stage that can be screened for and managed, which further complicates diagnosis and adds to the urgency of preventing OPC before it initiates and progresses [3].

### HPV infection

Over 90% of HPV-associated OPCs are caused by HPV16 and after first exposure at least two years passes before cancer develops, however it can often occur significantly later, with a mean timing of 35 years [4]. OPC due to HPV usually occur in a younger and healthier demographic as opposed to non-HPV associated OPC, which tends to occur in smokers and those who consume significant quantities of alcohol [5]. The amount of oral sex partners is the major risk factor associated with OPC development [1, 2]. The proportion of HPV-positive individuals was estimated at 6.9% in an American study of individuals aged between 14 to 69 years, with more men than women being positive for HPV (10.1% versus 3.6% respectively) [4]. This correlates with the consensus that there is increased incidence of HPV-associated OPC in men [2, 6, 7].

By comparison, many nations with HPV-vaccination programmes only target women due to the more widely known, and robust link between HPV-positivity and cervical cancer [8, 9]. Cervical cancer has the pre-cancerous stage of carcinoma-*in-situ (CIN),* which can be detected with screening and prevented with HPV vaccination [3]. OPC, by contrast, has no identifiable pre-cancerous stage and some have postulated that oral HPV-positivity may be the oropharyngeal equivalent to CIN and thus advocate strongly for its early elimination to prevent OPC occurrence [3, 10].

### HPV-vaccination

HPV-vaccination has been identified as a convenient and cost-effective way of reducing incidence of HPV-related OPC^,^ [11]. Most HPV-vaccination programmes largely target women, despite men being disproportionately affected by OPC [11].

Well-designed studies are lacking given the relatively recent implementation of HPV-vaccination and the late development of HPV-associated OPC (which occurs at a mean age of between 40-60). Studies have noted that HPV vaccines have been shown to reduce oral infection with HPV16. Oral HPV16 has been used in previous studies as a marker of OPC risk and its elimination as evidence of protection against HPV-associated OPC [12]. The FDA approved Gardasil (which targets HPV-6, 11, 16 and 18) as prophylaxis against HPV-related OPC in June 2020 [13]. Other countries, such as Australia, offer Gardasil free of charge to both boys and girls, through the National Immunisation Programme since 2013 and 2007 respectively [2].

The argument for pangender HPV vaccination has been countered in certain countries by querying cost-effectiveness and the potential ability to achieve herd-immunity through female vaccination only [14, 15]. These arguments do not take into consideration men-who-have-sex-with-men or that ‘gender-specific’ vaccination has markedly lower effectiveness than pangender vaccination against HPV [16, 17]. The case for male vaccination against HPV could be strengthened by a detailed examination of HPV-vaccination’s role in combatting OPC.

### Aim of review

This review aims to explore whether it is beneficial to undertake pangender HPV-vaccination to prevent HPV-associated OPC, focusing specifically on men. While HPV-vaccination has been shown to prevent cervical cancer, there is still a research gap surrounding HPV-vaccination and its role in preventing HPV-associated OPC, as fewer men have been vaccinated than women [1, 4, 18].

This study aims to provide recommendations for pangender HPV-vaccination to reduce HPV-associated OPC in men. This study is significant, as showing a relationship between these two variables could lead to international practice change, resulting in pangender vaccination, to prevent HPV-associated OPC.

Accordingly, the research question of this review is: how effective is HPV vaccination (intervention, comparator: unvaccinated) in preventing oropharyngeal cancer (outcome) in men (population)?

The null hypothesis is that HPV vaccination has no effect on prevention of oropharyngeal cancer in men.

The alternative hypothesis is that HPV vaccination has an effect on the prevention of oropharyngeal cancer in men.

### Literature search

To ensure that various sources were captured, the Ovid Medline, ProQuest Central and the Scopus databases were searched, including studies published between January 2017-present, for articles in the English language. The Cochrane Central Register of Controlled Trials and the Cochrane Database of Systematic Reviews were included from January 2005-present, to identify grey literature or ongoing clinical trials.

The searches were carried out on 22nd October 2021. See "Appendix 2" for search strategies and key words for each database. Inclusion criteria were studies that had full text available; were written in English; were related to cancer; were related to HPV; and were published from 2017 to present.

Exclusion criteria included studies that were non-systematic reviews (e.g., review articles, case reports); had no vaccination intervention; included a qualitative analysis only; did not include men in the study; had no mention of OPC; did not show overall prevalence of HPV; had no comparator group; and had no relevant or original data.

The search strategy was separately carried out by two authors (P.M. and E.M.). These two reviewers worked independently using the screening software Covidence, to screen title and abstracts for the first screening and then full text articles for the second screening, to determine articles for inclusion in this review [19]. All disputes were resolved by another independent author (S.D.).

### Assessment of risk of bias

The Cochrane Risk of Bias tool 2 (RoB-2) was employed to evaluate the included randomised controlled trial [20]. This tool was deemed appropriate to evaluate the strengths and weaknesses of included RCTs and has been validated extensively. One reviewer P.M. conducted the risk assessment. Each item was rated as “high”, “low” or “unclear” risk of bias. The ROBINS-1 tool was used to assess risk of bias for the non-randomised controlled trial and is another extensively externally validated tool [20].

### Assessment of quality

The National Institute of Health (NIH) quality assessment tool for observational cohort and cross-sectional studies and the NIH quality assessment tool for systematic reviews evaluated the quality of the observational studies and systematic reviews respectively [21]. By examining various aspects of the study design, a value of good, fair, or poor was allocated. The results of these are described later in the "Results" section.

## Main text

### Results

**Fig. 1:**
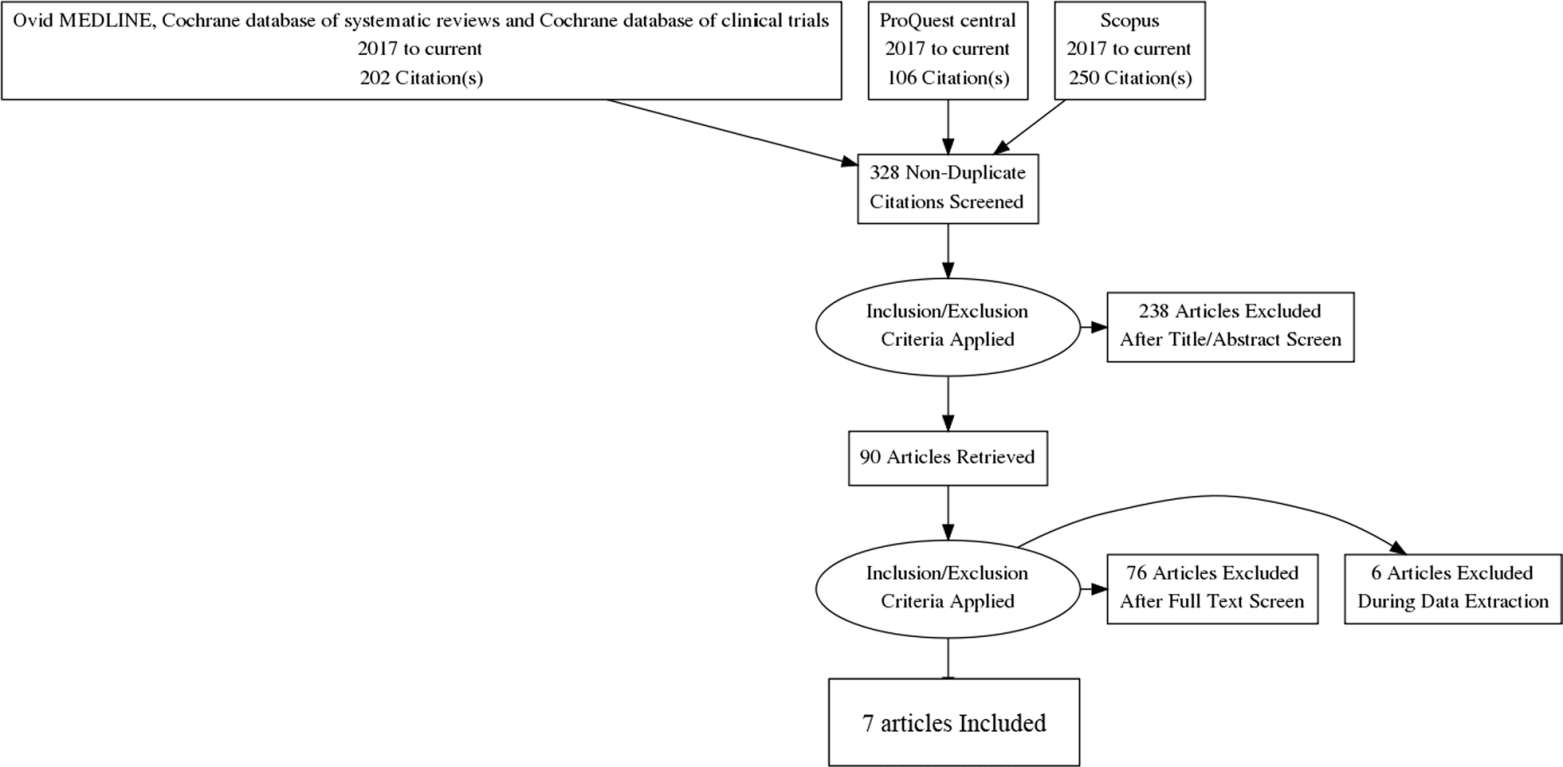
Preferred Reporting Items for Systematic Reviews and Meta-Analyses (PRISMA) statement [22]

The results of the literature search are presented as Fig. 1 (PRISMA diagram). The initial literature search generated 468 references. 328 studies were left for abstract screening after duplicated articles were removed. By dual-screening abstracts P.M and E.M, through the application of the inclusion and exclusion criteria, excluded 238 studies and 90 studies remained to be reviewed in full. Following the full text review, 76 articles were excluded, and a further 6 articles were excluded during data extraction due to the articles not meeting the inclusion criteria. Most excluded articles either did not include men, did not provide data on HPV-positivity or vaccination status, or were inappropriate study types (i.e., had low level evidence and rigour).

For the final inclusion, 7 articles remained. All 7 articles were published in English from 2017–2021. The included studies comprised 2 systematic reviews, 2 cross-sectional studies, 1 randomised controlled trial, 1 pre-post study with no control group and 1 prospective cohort study. Study characteristics can be seen as Table 1 and in more detail in “Appendix 1”. Risk of bias and quality assessment outcomes as described by the critical appraisal tools, are also summarised below.

**Table 1:** Study characteristics summary

Study	Study type	Characteristics	
		Number of participants	Outcome measure
Chaturvedi et al. [23]	Cross sectional study	2627	Infections in vaccinated (0%) versus unvaccinated men (2.13%)
Jamieson et al. [24]	Prospective cohort study	910	HPV positivity in saliva rose 34% to 44% at 1 year
Katz et al. [25]	Cross section study	3013 had a history of oropharyngeal cancer and 7732 were HPV vaccinated@@Of those with oropharyngeal cancer 1 had received the HPV vaccine and 3012 had not	Risk ratio increase of 23.8 (P = 0.0015) of developing oropharyngeal cancer in the male subgroup
MacCosham et al. [26]	Randomised Controlled Trial	167 couples so far	No results as yet
Nielsen et al. [27]	Systematic Review	48,777	Relative Prevention Percentage (RPP) of 83.9% following vaccination, from the cross-sectional studies as well as an RPP of 82.4% from the included randomised controlled trial and 83% in the longitudinal cohort study29
Parker et al. [28]	Single arm intervention trial	150	93.2% had oral anti HPV16 antibodies seven months post HPV vaccination
Tsentemeidou et al. [13]	Systematic Review and meta-analysis	13,285	Those who had received HPV vaccination were had an 80% decreased risk of having oral HPV16 compared to unvaccinated individuals (P < 0.0001)

### Study characteristics

Chaturvedi et al. carried out a cross-sectional study which showed significantly (P=0.007) decreased levels of oral HPV infections in vaccinated (0%) compared to unvaccinated men (2.13%) [23]. These researchers obtained data from 2627 men and women in the US National Health and Nutrition Examination Survey 2011-2014, describing their oral HPV status and the HPV-vaccination status. This study noted however, that due to low uptake overall among men, the effect of HPV-vaccination on oral HPV-positivity was 17% at a population level, but only 6.9% in men, highlighting the need for further uptake of HPV-vaccination in men.

An ongoing prospective longitudinal cohort study into Indigenous Australians by Jamieson et al., demonstrated evidence of HPV-vaccination in only 8.3% of participants, but did not distinguish between men and women [24]. These researchers followed 910 individuals over 12 months obtaining data on HPV-positivity, HPV-vaccination as well as various other healthcare related data. They noted that 3.3% of men were positive for oral HPV16/18 and this increased to 3.9% at 12 months follow up. Unfortunately, this study did not distinguish between vaccinated and unvaccinated individuals when describing HPV-positivity, thus it is unclear whether lack of vaccination led to this percentage increase.

Katz et al. used a cross-sectional study format and interrogated the correlation of OPC with HPV-vaccination using hospital databases [25]. They found a relative risk ratio increase of 23.8 (*P* = 0.0015) of developing oropharyngeal cancer in the male subgroup if they were not vaccinated. Out of 607,322 men, 3013 had previously had OPC and 7732 were HPV vaccinated. Of those with oropharyngeal cancer 1 had received the HPV vaccine and 3012 had not, suggesting that HPV vaccination could have reduced the incidence of OPC in this group.

An ongoing randomised controlled trial by MacCosham and colleagues is currently investigating whether HPV-vaccination can prevent HPV transmission among HPV discordant heterosexual couples [26]. They have so far recruited 167 couples and although the group have not reported any preliminary results yet, it appears to be a comprehensive and useful study capturing oral HPV results at baseline, and over the course of five follow up visits spanning one year, following randomisation to Gardasil or placebo. Similarly, HPV positivity would be an indication of OPC incidence in the future. While the results are pending, the inclusion of this study was appropriate in this systematic review, given its relevance of the subject matter to the question at hand.

A systematic review was published by Nielsen et al. highlighting the effect of HPV-vaccination on oral HPV-positivity [27]. They included 9 relevant studies from the past 5 years, comprising of 48,777 participants, and found a significant decrease of oral HPV-positivity in those immunised with HPV-vaccinations in multiple studies and heterogenous populations. They reported a mean Relative Prevention Percentage (RPP) of 83.9% following vaccination, from the cross-sectional studies, an RPP of 82.4% from the included randomised controlled trial and 83% in the longitudinal cohort study.

Parker et al., in a single arm intervention trial, measured oral HPV16/18 antibodies at multiple time points post HPV-vaccination in men aged between 27 and 45 years [28]. They found that 93.2% and 72.1% had HPV16 and 18 antibodies respectively, detectable in oral gargles 7 months post HPV-vaccination. The use of antibody levels to predict the potential development of HPV-associated OPC is a novel approach which differs from the other included studies who focus on oral HPV and this will be discussed further below.

In an important study, Tsentemeidou et al. undertook a systematic review and meta-analysis examining the link between oral HPV-positivity and HPV-vaccination, particularly pertaining to the risk of developing OPC [13]. They included 4 studies in the meta-analysis (N=13,285). Unfortunately, these papers did not distinguish between men and women in the HPV data in these studies which meant they were not suitable for inclusion in this systematic review. This meta-analysis overall showed that those who had received the vaccine had 80% less chance of having oral HPV16 compared to unvaccinated individuals (*P* < 0.0001). The authors also argued for oral HPV16 positivity to be a surrogate marker for future risk of developing OPC.

The included studies have given rise to the following themes: a link between HPV and OPC; a potential benefit to the vaccination of men to reduce rates of OPC and the likely cost-effectiveness of pangender HPV-vaccination [13, 23–29]. These themes will be discussed further below.

### Assessment of risk of bias

**Fig. 2:**
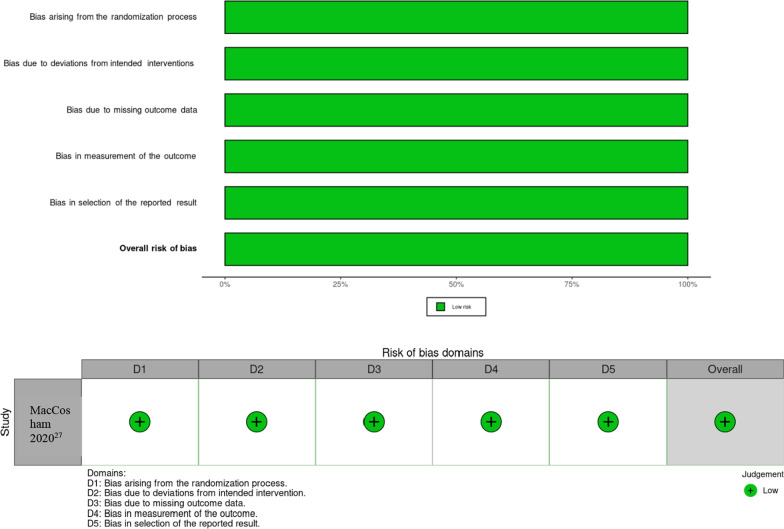
Cochrane Risk of Bias tool 2 assessing the MacCosham et al. paper (RoB-2) for the five risk of bias domains [20, 26]

**Fig. 3:**
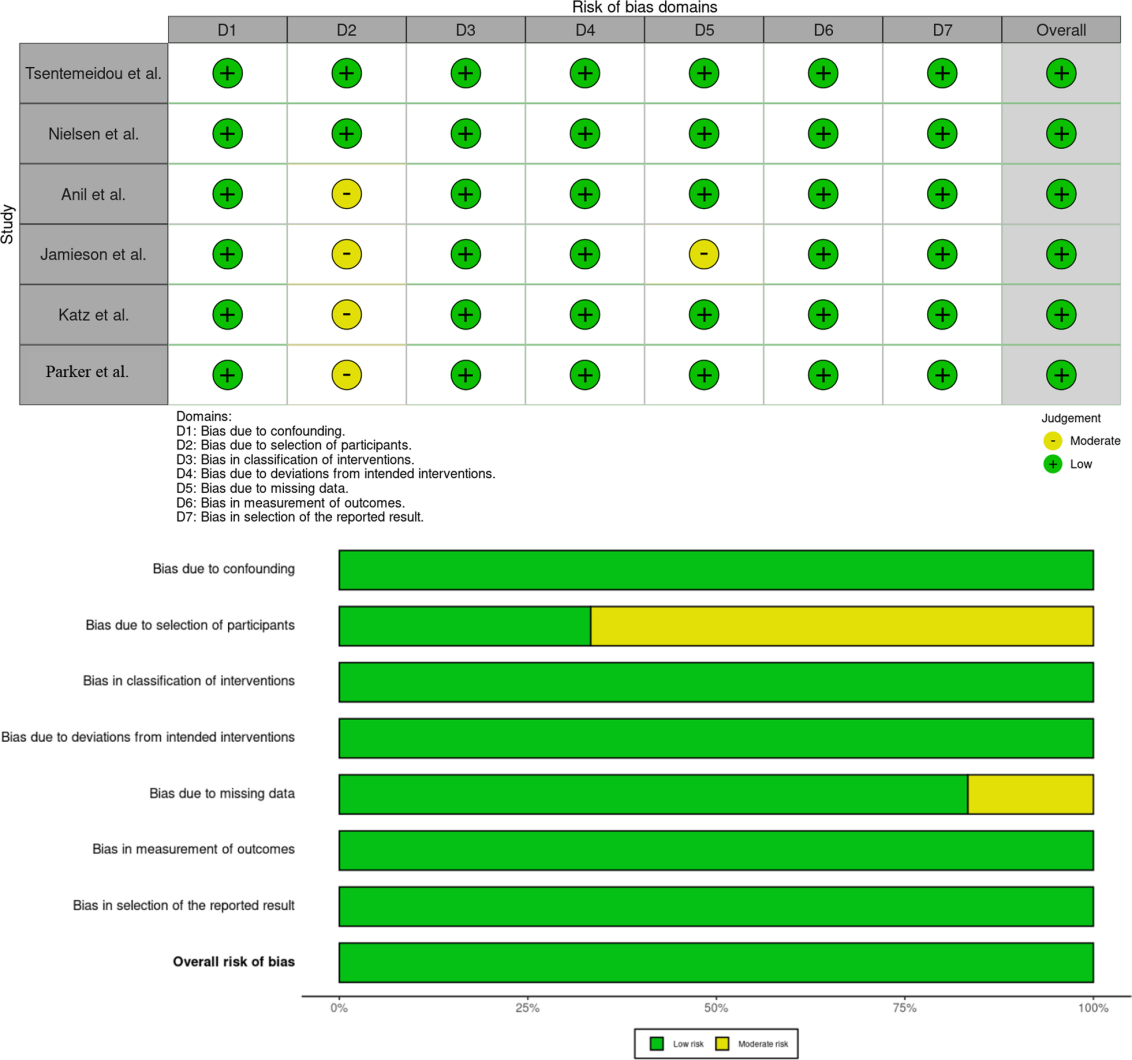
ROBINS-1 tool for assessing the Tsentemeidou et al., Nielsen et al., Chaturvedi et al., Jamieson et al., Katz et al., and Parker et al. for the seven risk of bias domains [13, 23–25, 27, 28]

A Cochrane risk of bias assessment was performed on the randomised controlled trial by MacCosham et al. using the RoB-2 tool [20]. The overall assessment of bias was determined by author P.M. as “low risk of bias”. A summary of the assessment can be found in Fig. 2. The ROBINS-1 tool was used to assess risk of bias for the non-randomised controlled trial studies. These 6 studies achieved a rating of low risk of bias, except for the study by Jamieson et al. which achieved a rating of low-moderate risk of bias. This was largely due to non-blinding during participant selection and bias due to missing data as the loss of follow up was greater than 20%. The results of this assessment can be found at Fig. 3.

### Assessment of quality

For the three systematic reviews, the NIH Quality Assessment of Systematic Reviews and Meta-Analyses determined quality and can be found at "Appendix 3". The systematic reviews all achieved a quality rating of good. The NIH Quality Assessment Tool for Observational Cohort and Cross-Sectional Studies was used as well as the Quality Assessment Tool for Before-After (Pre-Post) Studies with No Control Group [21] for the non-randomised studies. These all achieved a score of “good”. These results can be found in "Appendices 4 and 5".

## Discussion

This review focused primarily on synthesising all current evidence relating to the HPV-vaccination of men to prevent OPC. Overall, this study found that men who had received HPV-vaccination were significantly less likely to have or develop oral HPV16 in the future, with an average follow-up period of 12 months, compared with unvaccinated men, suggesting by proxy, a reduction in the incidence of HPV-associated OPC. Long term observational studies over the next 30–40 years, will reveal the impact of HPV-vaccination on OPC occurrence. Moreover, these observations of reduced HPV incidence, appear to be consistent regardless of study design or population.

### The challenges of diagnosing oropharyngeal cancer

Many of these studies used HPV positivity as a surrogate endpoint for HPV-associated OPC [25–27]. The link between HPV16 and oropharyngeal has been previously suggested by many studies [7, 16, 23, 30]. One example is a nested case control study showing a median time of onset of OPC of 3.9 years from detection of oral HPV16 positivity [30]. The use of oral HPV16 as a marker for OPC has been used by several groups and is a reasonable and necessary indicator that should provide enough evidence to demonstrate vaccine efficacy in preventing HPV16, and by extension OPC for the purposes of research and resource allocation [8]. In the study by Parker et al. HPV antibody positivity was used as a surrogate marker of protection against cancers such as OPC and represents a novel approach to examining this relationship between HPV-vaccination and OPC incidence. Long-term follow up will assist with determining the effectiveness of HPV antibodies as an indicator of immunity against HPV-associated OPC [28].

### Cost effectiveness, risks, and benefits of HPV vaccination

All of the studies agreed that while data may be lacking in the exact pathogenesis of HPV- associated OPC, the use of pangender HPV-vaccination was beneficial both from a cost-effectiveness standpoint as well as a risk/benefit standpoint, particularly in higher income countries [13, 23–29]. While some have argued that men may be sheltered from HPV-related sequelae such as OPC with herd immunity, data suggests that 80–90% of women would have to receive the HPV vaccine to achieve this, which is likely unattainable, particularly in low and middle income countries [13]. This also doesn’t consider the highly at-risk group of men-who-have-sex-with-men. The studies included bore out the idea that the most cost-effective and logical solution to the growing prevalence of HPV-related OPC is to advocate for a pangender HPV-vaccination strategy globally.

In relation to the Australian context, this notion of pan gender vaccination appears to be particularly important in ethnic minorities such as Indigenous Australians, whom in general have lower levels of health literacy and higher rates of oropharyngeal cancer compared to the general population [24]. Their data on men with HPV in Indigenous populations was particularly fascinating and painted a picture of higher levels of HPV than the general population and thus a likely higher risk for OPC development [24]. A significant limitation of this study is the fact that they didn’t separate men and women, so it was hard to interpret findings in our population group. The development of strategies to vaccinate men in such at risk groups needs to be a key priority, even in countries like Australia that already have a pangender HPV-vaccination programme, in combatting OPC development [24].

The systematic reviews by Tsentemeidou et al. and Nielsen et al. explore a similar question to this review however the key difference was that this review focused solely on men. While this led to a more focused evaluation, the downside is the lack of a detailed exploration of data relating to men in many HPV studies which led to certain studies included in the Tsentemeidou et al. and Nielsen et al. studies being excluded from our analysis. Both systematic reviews concurred with my overall findings that HPV-vaccination was likely linked to less risk of the occurrence of HPV-associated OPC however they did not focus solely on men.

There is currently a lack of long term follow up data available regarding men and HPV-positivity following vaccination [26]. Moreover, the presentation of the outcomes, varied significantly between studies. This is a potential area of future research that will be extremely beneficial to explore in depth in the form of a randomised controlled trial or future meta-analyses once more male-specific vaccination data becomes available.

The effect estimates vary across the included studies but overall, the studies were of a high quality and they are all in agreement that the use of HPV-vaccination in men is linked to desirable outcomes in terms of HPV-positivity or markers of oropharyngeal cancer development.

Overall, the results of this review suggest that there is a need for pangender HPV-vaccination in combatting oropharyngeal cancer in men. However, more work and observation time is required to definitively show that HPV-vaccination reduces the rate of OPC development in men.

## Conclusions

In conclusion, this review highlights the significant role to be played by pangender HPV-vaccination in the future battle against OPC. However, given the recent adoption of male HPV-vaccination and the latency of disease onset, the effects of male HPV-vaccination on OPC incidence remain to be seen over the next 30–40 years. As incidence of HPV-associated OPC increases, the use of oral HPV16 positivity as a surrogate marker for possible OPC development has become a key determinant in the efficacy of HPV-vaccination. In the included studies a significant decrease was detected overall in oral HPV in those vaccinated against HPV across heterogeneous populations and myriad study designs.

Further well-designed randomised controlled trials investigating the effects of vaccinating men for HPV and the impact of this on OPC development would be particularly helpful. Based on these findings it would be reasonable to suggest the benefit of vaccinating men as well as women against HPV to reduce the future prevalence of HPV-associated OPC and it seems reasonable that governments should take any opportunity to implement a pangender HPV-vaccination strategy.

## Data Availability

All data generated or analysed during this study are included in this published article (and its supplementary information files).

## Appendix 1: Study characteristics

**Table:**

Authors	Title	Year of publication	Country of publication	Study period	Study design	Research aim/question	Study population	Duration of follow-up	Vaccine/ comparator	Number of participants	Key findings
Chaturvedi et al. [23]	Effect of Prophylactic Human Papillomavirus (HPV) Vaccination on Oral HPV Infections Among Young Adults in the United States	2018	United States	2011–2014	Cross-sectional study	Investigating the levels of oral HPV-positivity in relation to HPV vaccination status	US adults from 18–33 with a clear vaccination history and oral HPV data	None	Yes	2627	There was less oral HPV16 positivity those who had been vaccinated against HPV compared to those who had not (0.11% v 1.61%; Padj = 0.008
Katz et al. [25]	The impact of HPV vaccination on the prevalence of oropharyngeal cancer (OPC) in a hospital-based population: A cross-sectional study of patient’s registry	2020	United States	2011–2020	Cross-sectional study	To analyse the interrelations between those with oropharyngeal cancer who have presented to hospital and their vaccination status	Hospital inpatients and outpatients in Florida over the 9-year period	None	Yes	1310334	Those without a history of HPV vaccintion were 19 times more likely to develop oropharyngeal cancer as opposed to those who were (RR 19.3657, 95% CI 7.2655 to 51.6177, *P* = 0.0001)
MacCosham et al. [26]	Transmission reduction and prevention with HPV vaccination (TRAP-HPV) study protocol: a randomised controlled trial of the efficacy of HPV vaccination in preventing transmission of HPV infection in heterosexual couples	2020	Canada	2014-present	Randomised Controlled Trial	To see if HPV vaccination can reduce transmission of oral HPV between HPV discordant partners	Sexually active heterosexual couples in Montreal from 18–45	12 months	Yes	167 couples so far	No results as yet
Nielsen et al. [27]	The Effect of Prophylactic HPV Vaccines on Oral and Oropharyngeal HPV Infection—A Systematic Review	2021	Switzerland	2016–2021	Systematic Review		Studies investigating the impact of HPV vaccines on oral or oropharyngeal HPV infection were enrolled	None	Yes	9 studies (48777 participants)	Analysis of the studies identified a significant reduction of oral HPV in those with a history of HPV vaccination
Parker et al. [28]	HPV-specific antibodies at the oral cavity up to 30 months after the start of vaccination with the quadrivalent HPV vaccine among mid-adult aged men	2019	United States	Not available	Interrupted time series study	To examine the link between OPC and HPV vaccination	Men ages between 27–45 in Tampa, FL and Cuernavaca, Mexico	24 months	No	150	All created HPV-16 antibodies and most had antibodies in oral gargles after seven months (HPV-16: 93.2%)
Tsentemeidou et al. [13]	Human Papillomavirus Vaccine to End Oropharyngeal Cancer. A Systematic Review and Meta-Analysis	2021	Greece	2020	Systematic Review and meta-analysis	To determine if HPV vaccination may decrease the incidence of oral HPV and OPC	Articles dealing with HPV vaccination and oropharyngeal cancer	None	Yes	6 studies (15240 participants)	A meta-analysis of 4 studies (1 RCT and 3 cross-sectional studies) saw a 80% (risk ratio, 0.20; 95% confidence interval, 0.09–0.43) lower risk of oral HPV16 infection in those vaccinated against HPV (P < 0.0001)

## Appendix 2: Search strategies and keywords

Ovid medline search strategy

**Table:**

#	Query	Results from 25 Oct 2021
1	((oropharynx or oropharyngeal or tonsil*) adj3 (cancer* or neoplasm* or carcinoma* or adenocarcinom* or tumour* or tumor* or malignan*)).mp. [mp = ti, ot, ab, sh, hw, kw, tx, ct, nm, fx, kf, ox, px, rx, an, ui, sy]	14,828
2	(human papilloma virus* or human papillomavirus* or HPV).mp. [mp = ti, ot, ab, sh, hw, kw, tx, ct, nm, fx, kf, ox, px, rx, an, ui, sy]	61,571
3	(vaccin* or Gardasil or Cervarix or silgard or 4vHPV or 2vHPV or 9vHPV).mp. [mp = ti, ot, ab, sh, hw, kw, tx, ct, nm, fx, kf, ox, px, rx, an, ui, sy]	445,330
4	(epidemiolog* or prevalence or incidence or mortality or death).mp. [mp = ti, ot, ab, sh, hw, kw, tx, ct, nm, fx, kf, ox, px, rx, an, ui, sy]	4,645,860
5	1 and 2 and 3 and 4	431
6	limit 5 to english language [Limit not valid in CDSR; records were retained]	396
7	limit 6 to yr = "2017 -Current"	202
**Database:**		
EBM Reviews—Cochrane Central Register of Controlled Trials < September 2021 >		
EBM Reviews—Cochrane Database of Systematic Reviews < 2005 to October 20, 2021 >		
Ovid MEDLINE(R) and Epub Ahead of Print, In-Process, In-Data-Review & Other Non-Indexed Citations and Daily < 1946 to October 22, 2021 >		

## Appendix 3: NIH quality assessment of systematic reviews and meta analyses [21]

**Table:**

NIH quality assessment tool for before-after (pre-post) studies with no control group [21]	Tsentemeidou et al. [13]	Nielsen et al. [27]
1. Is the review based on a focused question that is adequately formulated and described?	Yes	Yes
2. Were eligibility criteria for included and excluded studies predefined and specified?	Yes	Yes
3. Did the literature search strategy use a comprehensive, systematic approach?	Yes	Yes
4. Were titles, abstracts, and full-text articles dually and independently reviewed for inclusion and exclusion to minimize bias?	Yes	Yes
5. Was the quality of each included study rated independently by two or more reviewers using a standard method to appraise its internal validity?	Yes	Yes
6. Were the included studies listed along with important characteristics and results of each study?	Yes	Yes
7. Was publication bias assessed?	Yes	Yes
8. Was heterogeneity assessed? (This question applies only to meta-analyses.)	Yes	No
Overall Rating	Good	Good

## Appendix 4: NIH Quality Assessment tools for observational and cross-sectional studies [21]

**Table:**

NIH Quality Assessment Tool for Observational Cohort and Cross-Sectional Studies [21]	Chaturvedi et al. [23]	Jamieson et al. [24]	Katz et al. [25]
1. Was the research question or objective in this paper clearly stated?	Yes	Yes	Yes
2. Was the study population clearly specified and defined?	Yes	Yes	Yes
3. Was the participation rate of eligible persons at least 50%?	Yes	Yes	Yes
4. Were all the subjects selected or recruited from the same or similar populations (including the same time period)? Were inclusion and exclusion criteria for being in the study prespecified and applied uniformly to all participants?	Yes	Yes	Yes
5. Was a sample size justification, power description, or variance and effect estimates provided?	Yes	Yes	Yes
6. For the analyses in this paper, were the exposure(s) of interest measured prior to the outcome(s) being measured?	Yes	Yes	Yes
7. Was the timeframe sufficient so that one could reasonably expect to see an association between exposure and outcome if it existed?	Yes	Yes	Yes
8. For exposures that can vary in amount or level, did the study examine different levels of the exposure as related to the outcome (e.g., categories of exposure, or exposure measured as continuous variable)?	Yes	Yes	Yes
9. Were the exposure measures (independent variables) clearly defined, valid, reliable, and implemented consistently across all study participants?	Yes	Yes	Yes
10. Was the exposure(s) assessed more than once over time?	No	Yes	No
11. Were the outcome measures (dependent variables) clearly defined, valid, reliable, and implemented consistently across all study participants?	Yes	Yes	Yes
12. Were the outcome assessors blinded to the exposure status of participants?	No	No	No
13. Was loss to follow-up after baseline 20% or less?	Yes	No	Yes
14. Were key potential confounding variables measured and adjusted statistically for their impact on the relationship between exposure(s) and outcome(s)?	No	No	No
Overall Rating	Good	Good	Good

## Appendix 5: NIH Quality Assessment tools for studies with no control group. [21]

**Table:**

NIH Quality Assessment Tool for Before-After (Pre-Post) Studies With No Control Group [21]	Parker et al. [28]
1. Was the study question or objective clearly stated?	Yes
2. Were eligibility/selection criteria for the study population prespecified and clearly described?	Yes
3. Were the participants in the study representative of those who would be eligible for the test/service/intervention in the general or clinical population of interest?	Yes
4. Were all eligible participants that met the prespecified entry criteria enrolled?	Yes
5. Was the sample size sufficiently large to provide confidence in the findings?	Yes
6. Was the test/service/intervention clearly described and delivered consistently across the study population?	Yes
7. Were the outcome measures prespecified, clearly defined, valid, reliable, and assessed consistently across all study participants?	Yes
8. Were the people assessing the outcomes blinded to the participants' exposures/interventions?	No
9. Was the loss to follow-up after baseline 20% or less? Were those lost to follow-up accounted for in the analysis?	No, no
10. Did the statistical methods examine changes in outcome measures from before to after the intervention? Were statistical tests done that provided p values for the pre-to-post changes?	Yes
11. Were outcome measures of interest taken multiple times before the intervention and multiple times after the intervention (i.e., did they use an interrupted time-series design)?	Yes
12. If the intervention was conducted at a group level (e.g., a whole hospital, a community, etc.) did the statistical analysis take into account the use of individual-level data to determine effects at the group level?	N/A
Overall Rating	Good
